# Aberrant Development of Enteric Glial Cells in the Colon of Hirschsprung's Disease

**DOI:** 10.3389/fped.2021.746274

**Published:** 2021-11-05

**Authors:** Tingting Zhou, Wei Liu, Xiaofang Yu, Zengcai Cao, Weijing Mu, Peimin Hou, Chuantao Ren, Aiwu Li

**Affiliations:** ^1^Qilu Hospital, Cheeloo College of Medicine, Shandong University, Jinan, China; ^2^Department of Pediatric Surgery, Dezhou People's Hospital, Dezhou, China

**Keywords:** Hirschsprung's disease, enteric glial cells, enteric nervous system, glial fibrillary acidic protein, S100 calcium-binding protein B

## Abstract

**Objective:** The aim of this study was to explore the development of enteric glial cells (EGCs) in different segments of Hirschsprung's disease (HSCR).

**Methods:** Colonic specimens from 35 children with HSCR were selected to analyze the relative expression of glial fibrillary acidic protein and S100 calcium-binding protein B using Western blotting and real-time fluorescence quantitative PCR. Immunofluorescence and immunohistochemical staining were performed to determine the distribution of myenteric EGCs and neuronal cells in different segments of HSCR.

**Results:** There was a trend of diminished protein and mRNA expression of glial fibrillary acidic protein and S100 calcium-binding protein B from the proximal, dilated, and transitional segments to the aganglionic segment (*p* < 0.05). Immunofluorescence and immunohistochemistry showed that the EGCs in the aganglionic, transitional, and dilated colonic muscles were morphologically abnormal, which was consistent with the dysplasia of myenteric neurons.

**Conclusion:** Aberrant development of myenteric EGCs was observed in the colon of HSCR, which may affect the survival of enteric neurons.

## Introduction

The enteric nervous system (ENS) is the second largest nervous system in the human body, and it regulates intestinal movement, nutrient absorption, immune response, and other functions (1). During the embryonic period, the ENS originates from neural crest-derived cells and finally colonizes the distal colon to differentiate into enteric neural cells (ENCs) and enteric glial cells (EGCs) (2, 3). Defects during migration of neural crest-derived cells will cause the lack of ganglia in the distal colon to form aganglionic segments, which will lead to intestinal motor dysfunction diseases, such as Hirschsprung's disease (HSCR) and Hirschsprung allied disorders (4, 5). Several genes produced by EGCs, such as *glial cell-derived neurotrophic factor*, are involved in the survival, proliferation, migration, and differentiation of myenteric neurons (6, 7). Previous studies have found abnormal changes in ENCs, interstitial cells of Cajal, and EGCs in patients with slow colonic transit (8). As for the pathogenesis of HSCR, it is currently unknown whether abnormal changes in EGCs precede the missing of neurons.

Glial fibrillary acidic protein (GFAP), an astrocyte marker, is expressed in different subtypes of EGCs. It exists as a monomer in the human body and usually appears in mature EGCs (9). S100 calcium-binding protein β (S100β), which is highly expressed in activated glial cells, is also used as an astrocyte marker (9, 10). In order to study whether myenteric EGCs have developed abnormally in HSCR and to determine their distribution relationship with ENCs and their changing trends in HSCR, we compared the expression of GFAP and S100β in different segments of children with HSCR through several molecular biology methods.

## Materials and Methods

### Collection of Colon Specimens

Specimens were collected from 35 children with HSCR who underwent surgical treatment at the Pediatric Surgery Department of Qilu Hospital of Shandong University from December 2018 to December 2020, including 23 males and 12 females, with an average age of 16.15 ± 28.54 months. Among them, there were 30 cases of short-segment HSCR, 3 cases of long-segment HSCR, 1 case of total colonic type, and 1 case of extending to the small intestine. All research procedures were approved by the Ethics Committee of Qilu Hospital of Shandong University (KYLL-2018(KS)-092), and patient permission was obtained to use their specimens for research purposes only. Each colonic specimen was divided into four segments according to its lesion shape: aganglionic segment, transitional segment, dilated segment, and proximal segment. The aganglionic segment was defined to the narrow part lacking ganglia. The dilated segment was limited to the part that was significantly expanded due to feces accumulation, and the transitional segment was taken from the junction of the aganglionic and dilated parts. The proximal segment was collected from the relatively normal part of the resection margin. The aganglionic segment, transitional segment, and dilated segment were considered as diseased segments, and the proximal segment was used as control. Each part was no <500 mg. The tissue (0.5 cm) was soaked in 4% paraformaldehyde, and the remainder was stripped of the mucosa and submucosa (to eliminate the influence of hyperplastic cholinergic nerves) and stored at −80°C (11).

### Western Blot

WB was used to analyze GFAP and S100β protein expression in each segment. After thawing the tissue on ice, the Minute™ Total Kit (Invent, Plymouth, MN, USA) was used to extract total protein from the colon. Twenty micrograms of loading protein was electrophoresed on a 10% SDS-PAGE gel (Vazyme, Nanjing, China) and transferred to a PVDF membrane (Millipore, Germany). After blocking with 5% BSA, the primary antibody (1:1,000) was shaken overnight at 4°C. Then, the secondary antibody (1:5,000) was incubated for 1 h at room temperature. Detection was performed using chemiluminescence (Bio-Rad ChemiDoc). Supplementary Table 1 shows the details of the antibodies.

### Real-Time Fluorescence Quantitative PCR

RT-qPCR was used to analyze the mRNA expression of the targets. The tissues were thawed in RNAlater™ (Beyotime, Shanghai, China) on ice. An RNA Extraction Kit (Fastagen, Shanghai, China) was used to extract total RNA, and A260/A280 was between 1.8 and 2.2. The SYBR (Tokyo, Japan) reaction system was prepared after reverse transcription to cDNA, and a Roche Light Cycler 480 was used for RT-qPCR. Data were normalized to glyceraldehyde 3-phosphate dehydrogenase, and relative expression was calculated using the 2^−Δ*ΔCt*^ method. The primers were selected based on data published elsewhere or from the Beacon Designer software (Premier Biosoft, Palo Alto, CA, USA). The melting curve after each reaction was used to confirm specificity. The primer sequences are listed in Supplementary Table 2.

### Immunohistochemistry and Immunofluorescence

Immunohistochemistry (IHC) and IF were performed to compare the distribution of EGCs and ENCs in the diseased colonic muscles. The specimens were incubated in 4% paraformaldehyde and coated in 5-μm paraffin slices. After dewaxing and antigen retrieval, the slices were blocked with 5% BSA and incubated with the primary antibodies (1:500) overnight at 4°C, then incubated with IgG (H + L) or Alexa Fluor-594 and 498 secondary antibodies (Abcam, Cambridge, MA, USA) at 1:200 for 1 h at 37°C. All antibody incubation and washing steps were performed in PBS at pH 7.4. Images were acquired with an Olympus DP 72 (Tokyo, Japan) and the cellSens Dimension and Software image acquisition system. Diaminobenzidine (ZSbio, Beijing, China) staining was monitored under a microscope. Hematoxylin and 4′,6-diamidino-2-phenylindole were used for nuclear staining. ImageJ software was used for densitometric analysis of positive staining. Supplementary Table 1 shows the details of antibodies used.

### Statistical Analysis

All data are presented as means ± SD. GraphPad Prism 8.0 was used for statistical analysis and graphing. The significance of the differences between groups was calculated using one-way ANOVA, and *p* < 0.05 was considered to be statistically significant.

## Results

### GFAP and S100β Expression Diminished in Diseased Segments

**Figure 1:**
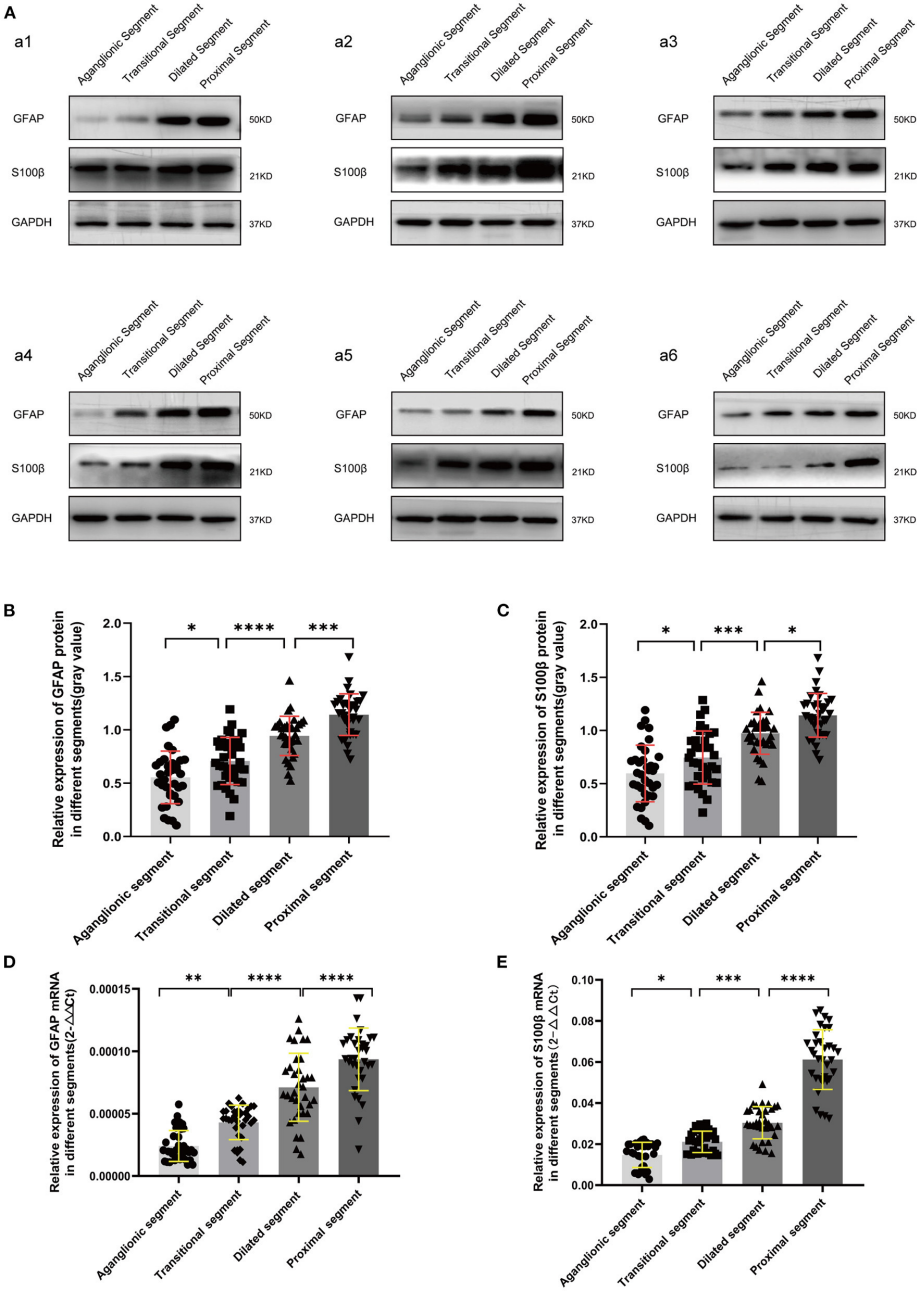
WB **(A-C)** and RT-qPCR **(D,E)** revealed significantly decreased protein and mRNA expression of GFAP and S100β in the diseased segments, compared with proximal segments of HSCR patients. Equal loading amounts were confirmed using glyceraldehyde 3-phosphate dehydrogenase. The assays were performed in triplicates, and values are given as mean ± SD. (ns, *p* > 0.05; *p < 0.05; **p < 0.01; ***p < 0.001; ****p < 0.0001).

The protein and mRNA expression levels of GFAP and S100β in the different segments were examined by WB and RT-qPCR. Compared with the housekeeping gene glyceraldehyde 3-phosphate dehydrogenase, there was a trend of decreased expression of GFAP and S100β from the proximal, dilated, and transitional segments to the aganglionic segment (*p* < 0.05) (Figure 1).

### EGC Hypoplasia and Loss of Neurons

**Figure 2:**
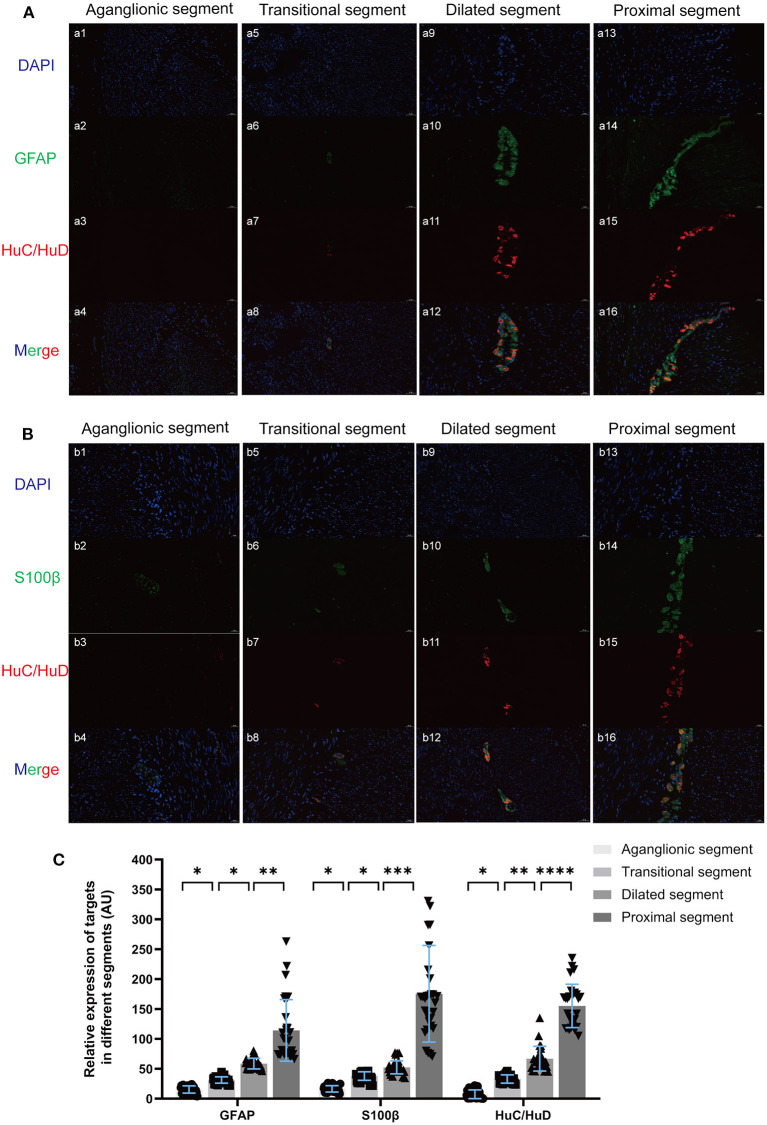
Double-label IF of GFAP (**A** green), S100β (**B** green), and HuC/HuD (red) revealed a corresponding relationship in the location of EGCs and ENCs in myenteric ganglions, and the average fluorescence intensity of the unit area was quantified **(C)**. There was no obvious positive staining of targets in the aganglionic segment (a1-a4, b1-b4). The shapes of ganglions in the muscles in transitional and dilated segments were irregular and hypoplasic (a5-a12, b5-b12), while the ganglion in proximal segments was more intact and the EGCs were located around the ENCs (a13-a16, b13-b16). (Scale bar: 20 μm ns, *p* > 0.05; *p < 0.05; **p < 0.01; ***p < 0.001; ****p < 0.0001).

GFAP, S100β, and the classic enteric neuron marker HuC/HuD were stained using IF, revealing a corresponding relationship between the development and location of EGCs and ENCs in myenteric ganglia (Figure 2). Attenuated ENCs and EGCs coexisted in diseased segments, and the EGCs of proximal segments were located around the ENCs and participated in the formation of myenteric ganglions.

### Expression Position of GFAP and S100β in Different Segments

**Figure 3:**
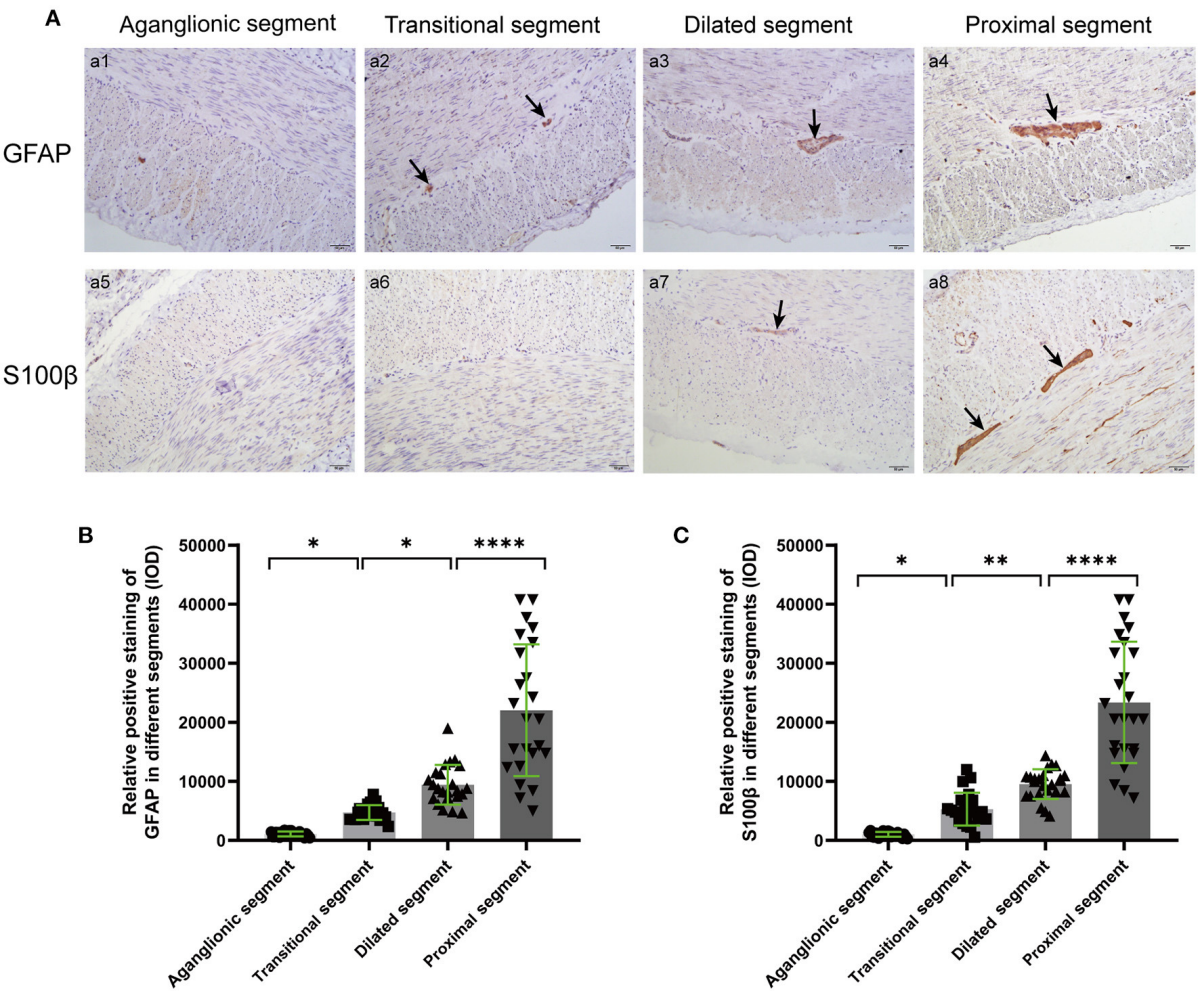
IHC **(A)** and quantitative analysis **(B,C)** of GFAP and S100β in the different segments of colonic muscle sections. The target GFAP and S100β were not significantly stained in aganglionic segments (a1, a5), and the positive staining particles in transitional (a2, a6 arrows) and dilated segments (a3, a7 arrows) were significantly attenuated and irregularly shaped, compared with the proximal segments (a4, a8 arrows). (Scale bar: 50 μm, ns, *p* > 0.05; *p < 0.05; **p < 0.01; ***p < 0.001; ****p < 0.0001).

The positions of GFAP and S100β in different segments were displayed by IHC, and positive staining was quantified based on intensity and area. The myenteric ganglia showed obvious positive staining of tan particles in proximal segments, and the positive particles of the aganglionic and transitional segments were significantly attenuated (Figure 3).

## Discussion

It was thought that EGCs were used to support neurons at first, while existing studies on EGCs focused more on inflammatory bowel diseases such as Crohn's disease and ulcerative colitis, and less on congenital diseases such as Hirschsprung allied disorders. EGCs are divided into different subtypes according to their location and function (12). On the one hand, they are similar to astrocytes in the central nervous system and can secrete a variety of neurotrophic factors to participate in the regulation of neuronal functions, such as glial-derived neurotrophic factor and S-nitrosoglutathione (6, 13, 14); on the other hand, EGCs also play a role in maintaining the balance of epithelial barrier and resist aggression through inflammatory reactions (7).

In this study, we compared the changing trends of myenteric EGCs in 35 children with HSCR and found their corresponding relationship with myenteric neurons in four segments. In line with the study by Tani et al. (15), in addition to aganglionic segments, immature EGCs appear in the proximal colon of HSCR children compared to normal controls. As a phenomenon-level study, the results are consistent with previous functional studies on EGCs, which found that the neurotrophic factor produced by EGCs can enhance the migration ability of neural crest-derived cells and augment the size of enteric neurospheres (16). Aubé et al. (17) confirmed that the absence of EGCs in the intestinal wall can lead to impaired colonic motor function. Soret et al. (10) showed that primary EGCs cultured *in vitro* enhance the barrier function of epithelial cells and produce Ca^2+^ transients upon induction of extracellular ATP. Therefore, we assume that abnormally developed EGCs in HSCR may lose their protective support and nutritional effect on ENCs.

However, after undergoing the surgery, some patients continued to experience postoperative complications, such as constipation soiling or enterocolitis, even though we thought that sufficient lesions had been removed. The appearance of persistent symptoms after surgery may be related to abnormalities in EGCs and ENCs in the unresected proximal colon (15, 18). This experiment prompted us to further study the interactions between EGCs and ENCs. Studies have shown that GFAP is related to delayed differentiation of EGCs. Under the stimulation of certain foreign antigens such as lipopolysaccharides, EGCs can proliferate reactively, exhibit an “active state” with GFAP upregulation, produce neuroprotective factors, and enhance the protective effect against ENCs (9, 13, 19). Carvalho et al. (20) proposed a more efficient preoperative biopsy method that recommends pathology mapping of the entire colon to assess the excision range. This prompted us to assume the possibility of preserving more colon tissue in future HSCR surgery by improving the function of neurons and EGCs in dilated and transitional segments. In addition, through the detection of GFAP and S100β, the normal EGCs in the resection margin may provide a reference for the scope of surgical resection (21). Furthermore, a reasonable assessment of ENS development can complement diagnosis and significantly reduce the incidence of complications such as postoperative enteritis (22).

An increasing amount of evidence demonstrated that EGCs play a vital role in maintaining the homeostasis of ENS. However, more attention was paid to enteric neurons in HSCR, compared with EGCs. Whether and how EGCs are involved in the development and maturation of ENS and whether hypoplasia of EGCs precedes the absence of neurons remain to be further studied.

## Data Availability Statement

The raw data supporting the conclusions of this article will be made available by the authors, without undue reservation.

## Ethics Statement

The studies involving human participants were reviewed and approved by Qilu Hospital, Cheeloo College of Medicine, Shandong University. The patients/participants provided their written informed consent to participate in this study.

## Author Contributions

TZ: design of the work, acquisition, analysis, and interpretation of data. WL, XY, ZC, WM, PH, and CR: collection of specimens and obtaining informed consent from patients. AL: drafting the manuscript or revising it critically for important intellectual content. All authors contributed to the article and approved the submitted version.

## Funding

This work was funded by the National Natural Science Foundation of China (Project nos. 81873846 and 82071682).

## Conflict of Interest

The authors declare that the research was conducted in the absence of any commercial or financial relationships that could be construed as a potential conflict of interest.

## Publisher's Note

All claims expressed in this article are solely those of the authors and do not necessarily represent those of their affiliated organizations, or those of the publisher, the editors and the reviewers. Any product that may be evaluated in this article, or claim that may be made by its manufacturer, is not guaranteed or endorsed by the publisher.
