# Supplementary material for: Aberrant Development of Enteric Glial Cells in the Colon of Hirschsprung's Disease
Source: Front Pediatr. 2021 Nov 5;9:746274. doi: 10.3389/fped.2021.746274 (PMC8602875; doi:10.3389/fped.2021.746274)
Supplement: Supplementary file 1 [file Table_1.DOCX]

**Suppl Table 1**. Antibodies used in this article

| Antibodies | Manufacturers | Sources | Cat. No | Lot. No |
| --- | --- | --- | --- | --- |
| GFAP | Novus, USA | Mouse | NBP1-05197SS | 090817 |
| S100β | Abcam, USA | Rabbit | ab52642 | GR3215095-15 |
| HuC/HuD | inInvitrogen, USA | Mouse | A21271 | 2105721 |
| GAPDH | CST, USA | Rabbit | 2118S | 14C10 |
| IgG (H + L) | Affinity, China | Goat | S0001 | 7427s83 |
| Alexa Fluor 488 | Abcam, USA | Goat | Ab150081 | GR3364096-1 |
| Alexa Fluor 594 | Abcam, USA | Goat | Ab150120 | GR3332661-2 |
