# Supplementary material for: Aberrant Development of Enteric Glial Cells in the Colon of Hirschsprung's Disease
Source: Front Pediatr. 2021 Nov 5;9:746274. doi: 10.3389/fped.2021.746274 (PMC8602875; doi:10.3389/fped.2021.746274)
Supplement: Supplementary file 2 [file Table_2.DOCX]

**Suppl Table 2**. Primer sequences used in this article

| Gene | Species | Forward | Reverse |
| --- | --- | --- | --- |
| GFAP | homo | GCTTTGCCAGCTACATCGAG | TGCCAGATTGTCCCTCTCAAC |
| S100β | homo | GGAGACGGCGAATGTGACTT | TCAAAGAACTCGTGGCAGGC |
| GAPDH | homo | GCACCGTCAAGGCTGAGAAC | TGGTGAAGACGCCAGTGGA |
